# Author Correction: Strain driven emergence of topological non-triviality in YPdBi thin films

**DOI:** 10.1038/s41598-021-95598-z

**Published:** 2021-08-03

**Authors:** Vishal Bhardwaj, Anupam Bhattacharya, Shivangi Srivastava, Vladimir V. Khovaylo, Jhuma Sannigrahi, Niladri Banerjee, Brajesh K. Mani, Ratnamala Chatterjee

**Affiliations:** 1 Department of Physics, Indian Institute of Technology Delhi, New Delhi, India; 2 Department of Mechanical Engineering, Indian Institute of Technology Delhi, New Delhi, India; 3 National University of Science and Technology “MISiS”, Moscow, Russia 119049; 4 Department of Physics, Loughborough University, Loughborough, LE11 3TU UK

Correction to: *Scientific Reports* 10.1038/s41598-021-86936-2, published online 06 April 2021

The original version of this Article contained an error in the Acknowledgments section.

“SS, NB and RC acknowledges the financial assistance received from SPARC proposal #754.”

now reads:

“SS, NB and RC acknowledge the financial assistance received from SPARC proposal #754 & DST Nanomission project (DST/NM/TUE/QM-11/2019).”

The original Article has been corrected.

